# Osteogenesis Improvement of Gelatin-Based Nanocomposite Scaffold by Loading Zoledronic Acid

**DOI:** 10.3389/fbioe.2022.890583

**Published:** 2022-04-25

**Authors:** Sayed Behnam Abdulahy, Mona Esmaeili Bidhendi, Mohammad Reza Vaezi, Mehrdad Moosazadeh Moghaddam

**Affiliations:** ^1^ Biomaterial and Tissue Engineering Department, Breast Cancer Research Center, Motamed Cancer Institute, ACECR, Tehran, Iran; ^2^ Faculty of New Science and Technology, University of Tehran (UT), Tehran, Iran; ^3^ Department of Nanotechnology and Advanced Material, Materials and Energy Research Center (MERC), Karaj, Iran; ^4^ Applied Biotechnology Research Center, Baqiyatallah University of Medical Sciences, Tehran, Iran

**Keywords:** zoledronic acid, halloysite, gelatin, nanocomposite, osteogenesis, scaffolds

## Abstract

Bisphosphonates (BPs) such as Zoledronic acid (ZA) are a subset of synthetic small molecules, which are now marketed as the main drugs to stimulate the growth and differentiation of osteoblast cells, thereby increasing bone formation as well as preventing bone loss. Also, Halloysite Nanotubes (HNTs)-polymer composites have attracted a lot of attention due to their high surface-to-volume ratio, low density, and high hydrophilicity, and are easily dispersed in hydrophilic biopolymers. In addition, their ability to carry enough amounts of drugs and the ability to control release has been demonstrated. Based on studies, the Gelatin-based scaffold with Halloysite nanotube (HNT) has the capacity as a drug carrier and Zoledronic acid (ZA) sustains release. Previous studies show that using ZA intravenously has some severe side effects and limitations. But by attention to the advantages of its osteogenesis, the current study has been done in order to reduce the side effects of local delivery of it. The 3-dimensional scaffolds were prepared by the Freeze-drying method. Characterization methods such as FE-SEM, FTIR, XRD, and release behavior of the scaffold has been performed to evaluate the features of the scaffolds. In fact, as-prepared Gel-HNT/ZA release 49% ZA in Phosphate Buffered Saline (PBS) within 21 days. The mechanical properties have been increased after adding HNTs and ZA from 10.27 to 26.18 MPa. Also, the water absorption has been increased after adding HNTs and ZA from 1.67 to 5.02 (g/g). Seeded human Adipose stem cells (hASCs) on the prepared scaffolds showed that the ZA effectively elevated the proliferation of the hASCs and also the MTT results proved the non-toxicity of all prepared scaffolds by high cell viability (˃80%). The osteogenic differentiation has been accelerated as displayed by ALP and Ca assay. The results propose that the HNTs-loaded Gelatin scaffold could control the releasing of ZA and its localized delivery at the defect site, simultaneously promoting the mechanical and osteogenesis ability of gelatin-based scaffolds.

## 1 Introduction

An important and big class of orthopedic problems is bone defects (Wiese and Pape, 2010; Guerado and Caso, 2017; Gage et al., 2018; Kim et al., 2020). Over the last few decades, many efforts have been made by researchers to find suitable bone replacements. Early efforts focused on the use of metal substitutes. However, corrosion of these implants in the patient’s body, in addition to their mechanical properties and gradual loosening, led to the release of highly toxic metal ions and subsequent inflammatory reactions of these products with the surrounding tissue (Parks and Lakes, 1992). Another problem with metals is their very high modulus (Liu and Dixit, 1997). The elastic modulus of metals is higher than 100 GPa, which will be much higher than the density of dense bone (6–20 GPa). The result of this high stiffness is the occurrence of the phenomenon of stress protection on the growing bone, which will lead to thinning of the new bone tissue and will increase the probability of its re-failure (Flahiff et al., 1996). Problems such as this have drawn the attention of many researchers to newer materials; Materials that do not have the problems of previous implants and at the same time allow the formation of bone tissue in the defect position with higher speed and quality. Thus, a new chapter called “Bone Tissue Engineering” was opened and new biomaterials for this purpose were introduced to the medical community (Park and Bronzino, 2000; Burg et al., 2000; Lee et al., 2001). It has been reported that the production of novel scaffolds as the carrier for osteogenesis drugs could be an advantageous approach to eschew systemic problems of the drug while improving its therapeutic efficiency (Cartmell, 2009; Xu et al., 2020).

Small molecules are natural or synthetic molecules that have low molecular weight and have the ability to regulate cellular, tissue, and therapeutic functions. These molecules are organic in nature and have less than 1,000 Da in size (Carbone et al., 2014). Bisphosphonates (BPs) are a subset of small molecules, small synthetic compounds, and now marketed as the main drugs to stimulate the growth and differentiation of osteoblast cells, thereby increasing bone formation as well as preventing bone loss.

BPs have been shown to reduce the risk of vertebral and non-vertebral fractures (Lambrinoudaki et al., 2006). They are pyrophosphate analogues that have been modified to act as a specific stimulant of osteogenesis and anti-bone resorption, the mechanism of which is to have a strong inhibitory effect on osteoclasts and increase bone induction. BPs have a strong affinity for bone surfaces and accumulate there, and due to this selective action, they have systemic side effects. BPs are also potent bone resorption inhibitors that inhibit osteoclast differentiation (Coxon et al., 2000) and stimulate the programmatic death of osteoclast cells (Benford et al., 2001). The results show a high tendency of BPs to bone mineralization and especially the return of lost bone volume (Nancollas et al., 2006). Using of bisphosphonates is considered, as one of the applicable methods that can be increased the integration of scaffolds with the surrounding bone tissue by increasing bone growth, for example, Zoledronic acid (ZA), which is a member of this group. (Yang et al., 2020).

In recent decades, Halloysite Nanotubes (HNTs)-polymer composites have attracted a lot of attention due to their high surface-to-volume ratio, low density and high hydrophilicity, and are easily dispersed in hydrophilic biopolymers (Ji et al., 2017; Zare and Rhee, 2021c; Zare and Rhee, 2021b). In addition, HNTs have shown excellent mechanical properties and good biocompatibility (Abdollahi Boraei et al., 2020a; Abdollahi Boraei et al., 2021a; Zare and Rhee, 2022). HNT-containing polymer nanocomposite scaffolds have been shown to be capable of carrying sufficient amounts of drugs as well as their controlled release (Zare et al., 2021). Also, the opposite charge on the outer and inner surface of HNTs makes them attractive for electrostatic bonding with polymers and drugs (Lee Y. J. et al., 2019; Zare and Rhee, 2021a).

In the present study, a gelatin based-nanocomposite with HNTs containing of Zoledronic acid was used for bone regeneration, with the aim of targeting ZA release in a controlled manner. This new drug delivery system is designed as a novel nanocomposite system for use in bone tissue engineering. Also, HNT was also used as a carrier of ZA drug, which strengthened the specificity of the system. Moreover, improving the physical, mechanical and bone properties of gelatin scaffolds has been expected by using this composition. The application of BTE is evaluated by making a specific scaffold in combination with gelatin. Gelatin was selected as a matrix of scaffold due to its non-toxicity, low cost, availability, ease of processing, high cell adhesion and proliferation (Ji et al., 2017). Finally, the effects of both HNT and ZA loading on the biological properties of gelatin-based scaffolds were evaluated *in vitro* and *in vivo*.

## 2 Materials and Methods

### 2.1 Loading of ZA on/into HNTs

At the first, HNTs (4 mg/ml; Sigma Co.) were added to ZA supersaturated solution (4 mg/ml; Sigma Co.) mixed in deionized water under vacuum situation. After 20 min, the suspension was removed, stirred vigorously overnight and then centrifuged for 20 min with 6,000 rpm. The obtained precipitates were washed thoroughly and then freeze dried.

### 2.2 Preparation and Characterization of Scaffolds

#### 2.2.1 Preparation

Gelatin (2.4 g; sigma, Type A) was poured in 40 ml of deionized water to be dissolved at room temperature and HNTs (4 wt. % relative to gelatin) and ZA-loaded HNTs were added to the solution of gelatin. After stirring thoroughly, the prepared suspensions were dispensed in 24 well tissue culture plates, kept in the refrigerator at 4°C for 24 h and finally frozen at –80°C overnight before doing freeze-drying process (OPERON Company, Korea). Moreover, pure gelatin scaffold without HNTs was chosen as the control group. The prepared scaffolds were coded as 1) Gel (without HNTs), 2) Gel/HNTs (without ZA), and 3) Gel/HNT-ZA (with ZA). Then, the cross linking of scaffolds was done in a sealed desiccator in the presence of glutaraldehyde (8 ml) at 37°C for 4 h. Afterward, the prepared scaffolds were washed with 1% (W/V) of *Glycine* solution to remove the unreacted glutaraldehyde.

#### 2.2.2 Field Emission Scanning Electron Microscopy

The ross-section views of the composite scaffolds were investigated by FE-SEM (S-4700 model, HITACHI Company, Japan). The dispersion of the ZA into the nanocomposite samples was evaluated by energy dispersive spectroscopy (EDS). The ImageJ software was hired to examined pore size and the porosity percentage of each scaffold from the three SEM images by the following equation (Shahriarpanah et al., 2016):

$$Porosity\left(\%\right)=\frac{A_{p}}{A_{T}}\times 100$$

In this equation, A_p_ is total area of pores in each cross-section and A_T_ is total area of each cross-section from the SEM images.

#### 2.2.3 Water Uptake

In order to examine the equilibrium water adsorption, the finalized scaffolds with the initial weight (W_o_) were place into the deionized water for 24 h at 37°C. Then, the samples were pulled out and the excess water was wiped off by using a filter paper and weighed (Ws). The equilibrium water adsorption was gained by the following equation (Abdollahi Boraei et al., 2021a; Zadegan et al., 2019):

$$\mathrm{Equilibrium}\;\mathrm{water}\;\mathrm{absorption}\left(g/g\right)=\frac{\text{Ws}-\text{W}0}{\text{W}0}$$

#### 2.2.4 Fourier-Transform Infrared

FTIR spectroscopy (Spectrum 100, PerkinElmer Company, United Kingdom) was hired to investigate the changes in the structure of gelatin after HNTs and ZA-loaded HNTs addition. X-ray diffraction analysis (XRD; D8 ADVANCE diffractometer, BRUKER Company, United Kingdom) was done by using Cu-Kα radiation at 40 kV and speed of 2°/min to evaluate the crystallographic changes of (GEL) scaffolds after HNTs and ZA-loaded HNTs addition.

#### 2.2.5 Mechanical Properties

The compressive behavior of the prepared scaffolds (GEL, GEL-HNT and GEL-HNT/ZA) were measured by the ZWICK/ROEL Z005 testing machine (ZWICK, Germany) with the cross-head speed of 1 mm/min.

### 2.3 Release Behavior of ZA

Drug release behavior of the scaffolds were measured by immersing and incubating the scaffolds in PBS at 37°C in a shaker incubator (90 rpm). The scaffold-released ZA level was examined by UV-Visible spectroscopy (NANODROP 2000c; Thermo Scientific Company, United States) at the wavelength of 210 nm at many times point (24, 48, 72 h and 7, 14 and 21 days).

### 2.4 Cellular Assays

#### 2.4.1 Cell Culture Procedure

The hASCs (Shariati Hospital, Tehran, Iran) were cultured in Dulbecco’s modified Eagle’s medium (DMEM/F-12; Gibco, United States) with 12% fetal bovine serum and 1% Pen-strep (Gibco, United States), followed by incubation at humidified condition with 5% CO_2_ at 37°C. Moreover, the osteogenic agents (10,000 µM Beta-Glycerophosphate, 50 μg/ml l-Ascorbic acid and 10^–4^ μM Dexamethasone) were added to the medium. The prepared medium must be changing every 3 days. All cellular assays were performed by the second-passage of cells. Before seeding the cells on the scaffolds, all scaffolds were sterilized by UV irradiation for 20 min on each side of them.

#### 2.4.2 Cytotoxicity of Prepared Scaffolds

Cytotoxicity of the scaffolds were measured by the 3-(4,5-Dimethylthiazol-2-yl)-2,5- Diphenyl Tetrazolium Bromide assay kit (MTT, Bioidea, Iran). The hASCs were seeded on sterilized scaffolds (20,000 cells/cm^2^). Then they were cultured in DMEM (15% FBS) at 5% CO_2_ and 37°C in the incubator for 1, 4 and 7 days. After that, 30 μl MTT was poured into each well, and incubation proceeded for 3 h. Then 200 μl DMSO (Bioidea, Iran) was added to cells and keep them for 30 min in a dark place. Finally, the absorption amounts were examined by using an ELISA plate reader (Stat Fax-2100, United States) at a wavelength of 545 nm.

#### 2.4.3 Osteogenesis Assays

Alkaline phosphatase (ALP) activity assay was performed by using 200 μl of RIPA buffer. The total protein was extracted from hASCs cultured on Tissue Culture polystyrene (TCPS) and different scaffolds after 7 and 14 days during the period of study. In order to sedimentation of cell debris, the lysate was centrifuged at 1,200 rpm at 4°C for 5 min. Then, the supernatant was collected and ALP activity was examined with an ALP assay kit (Parsazmun, Tehran, Iran).

#### 2.4.4 Calcium Assay

Calcium deposition on the scaffolds was examined by using Alizarin red staining (ARS) method. The hASCs cells were seeded on the scaffolds which were placed in the 24 well tissue culture plate. After 7 and 14 days of incubation, the fixation of cell-sample constructs was performed by the following instruction: 1) rinsing samples with PBS, 2) dehydration of samples through graded concentrations of ethanol 50, 70, 80 (for 15 min), 96, and 100% (for 8–10 min), respectively, 3) the Alizarin red-40 mM was poured on the samples and keep for 20–30 min. The deposited calcium, which is red/purple colour, was then observed using an inverted optical microscope (Nikon Eclipse TE2000-U, Japan).

### 2.5 Statistical Analysis

All tested were performed in triplicates and the data were presented as the mean ± standard deviation (SD). One-way ANOVA was used to evaluate the differences between the samples. Statistically significant levels were considered to be *p* < 0.05.

## 3 Results and Discussion

### 3.1 Characterization of the Nanocomposite Scaffolds

**FIGURE 1:**
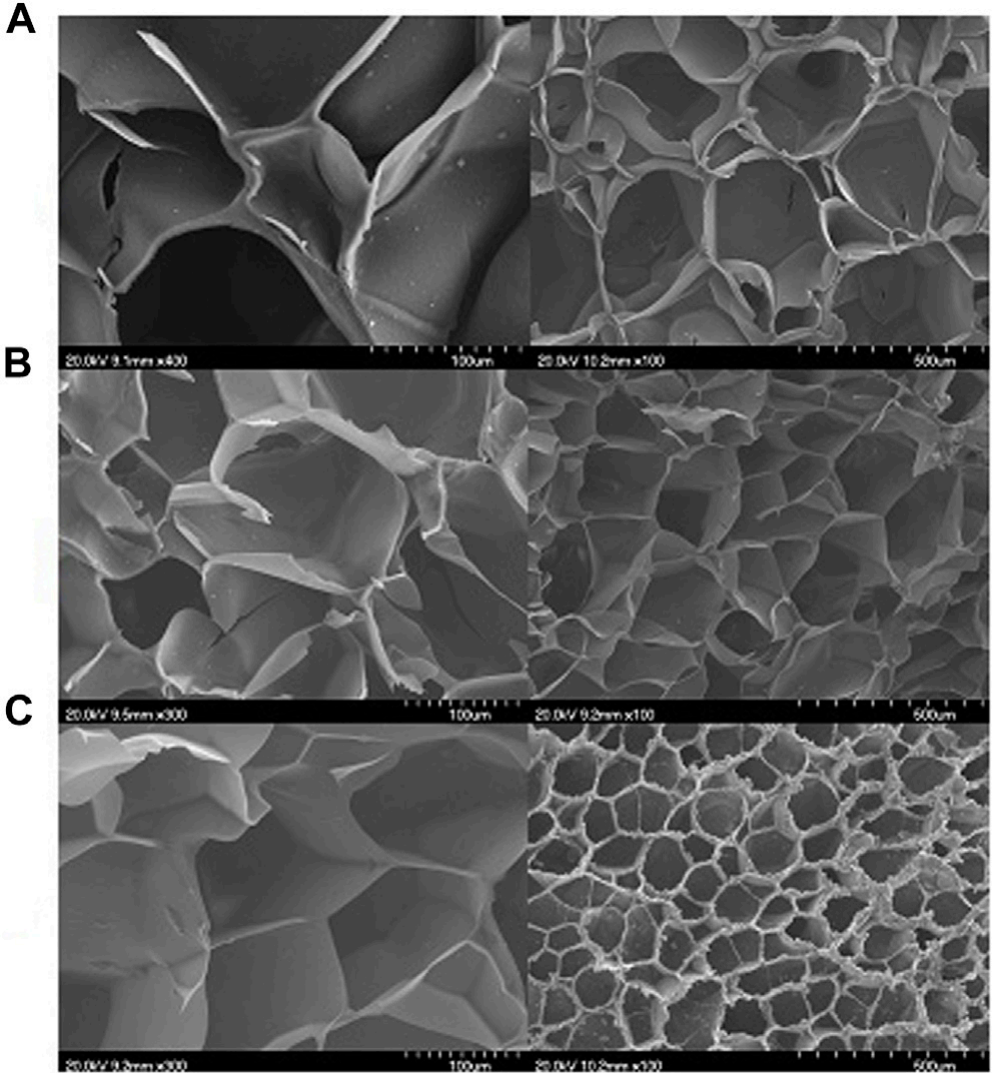
The morphology of the **(A)** Gel, **(B)** Gel-HNT and **(C)** Gel-HNT/ZA scaffolds.

The morphology and pore size of the prepared scaffolds are shown in Figure 1. As expected, an interconnected network structure was observed in the scaffolds that is in line with the our previous study in which a gelatin-based scaffold containing HNTs and the hydrophilic drug strontium ranelate was synthesized, and the results showed a uniform structure with well-sized and interconnected cavities (Abdollahi Boraei et al., 2020b). Since the pore size distribution doesn’t significantly change with the addition of ZA, the prepared scaffolds show almost similar surface morphologies which consisted of homogenous porosities.

The pore size results showed that the Gel scaffold has the minimum pore size with the mean value of 80.94 ± 43.80 µm, while the pore size elevated to 175.57 ± 21.09 µm in Gel-HNT and 257.89 ± 16.11 µm in Gel-HNT/ZA scaffolds. As expected, the augmentation of HNTs and HNT/ZA leads to create bigger pores, which is in a desire range for BTE applications. In the studies, different numbers have been reported for the appropriateness of the pore size of scaffolds in tissue engineering, for example, in the article of Li et al., The number of 300–400 microns has been selected as the appropriate range (Li et al., 2016; Rashedi et al., 2021). Lee et al. Also considered the number 500 microns to be suitable for adhesion, differentiation, and proliferation of cells inside the scaffold (Lee D. J. et al., 2019). In our studies reported that the scaffolds with larger pore size (>100 µm) provide better matrices for bone regeneration (Abdollahi Boraei et al., 2021b). In general, this number (porosity) should be suitable for delivering nutrients into the scaffold and removing waste from the scaffold (Tolba et al., 2010; Ghazalian et al., 2022).

Table 1 displays the porosity percentage of the prepared scaffolds. A similar upward trend is observed for porosity. Increasing pore size and porosity probably can effects on the electrostatic repulsion forces between carboxyl and hydroxyl groups in gelatin (Type A; pI = 7–9) and HNTs, since both HNTs and gelatin are negatively charged at pH 7 (Abdollahi Boraei et al., 2021a).

**TABLE 1:** Pore size of the scaffolds.

Sample	Gel	Gel-HNT	Gel-HNT/ZA
Pore size (µm)	80.94 ± 43.80	175.57 ± 21.09	257.89 ± 16.11

**FIGURE 2:**
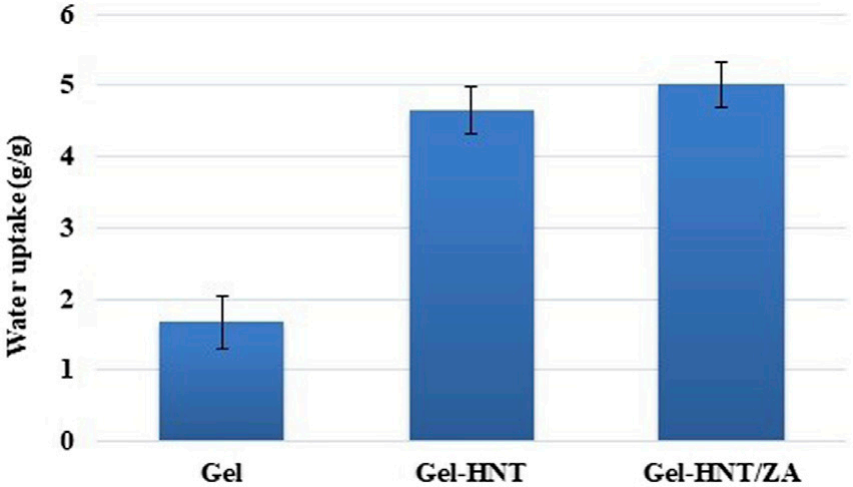
Equilibrium water uptake values of the scaffolds.

The equilibrium water uptake results of the scaffolds are brought in Figure 2. The water uptake for the Gel-HNT/ZA scaffold was the highest (5.02 ± 0.316 (g/g)), while this value for the Gel and Gel-HNT scaffolds decreased to 4.66 ± 0.33 and 1.67 ± 0.369 (g/g), respectively. This increase can be related to the larger pore nature in the Gel-HNT/ZA scaffold and proved by such studies that the microstructure can affect the swelling ratio (Mirahmadi et al., 2013). The more swelling ratio resulted in more water adsorption that is suitable for dipper diffusion of nutrition and better removing of waste from the matrix (Mirahmadi et al., 2013).

**FIGURE 3:**
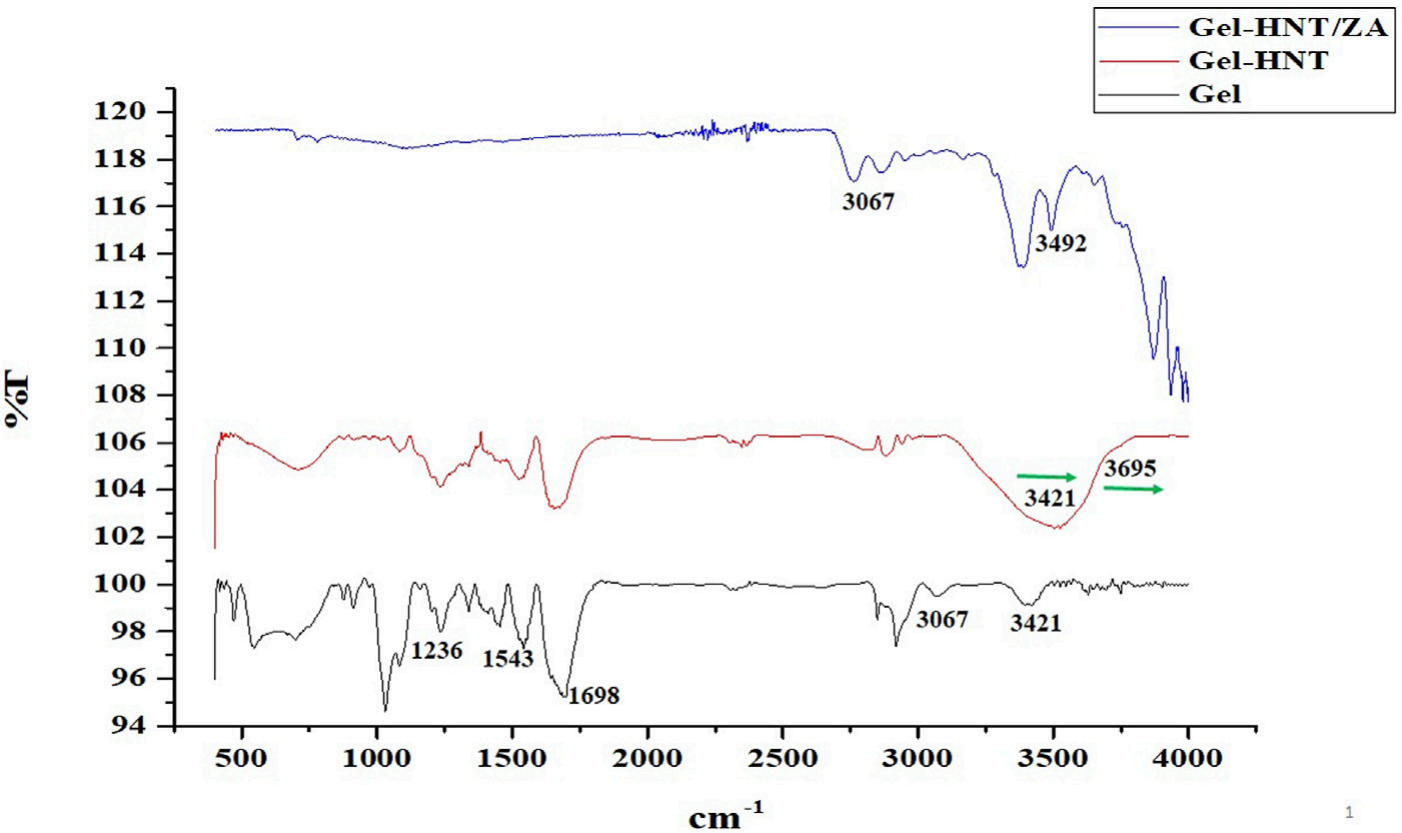
The FTIR spectra of the Gel, Gel-HNT and Gel-HNT/ZA Scaffolds.

FTIR analysis examines the chemical interaction of scaffold components that can determine the three-dimensional structure, the drug release properties, and the biological properties of the scaffold. Figure 3 shows the FTIR graphs of the scaffolds. As expected, in the gelatinous scaffold graph, characteristic gelatin peaks, including amide I, II, III, amide B, and amide A peaks, were observed at 1,698, 1,543, 1,236, 3,067, and 3,421 cm^−1^, respectively. In the previous study, where the gelatin-based scaffold was made by the freeze-drying method, the peaks mentioned above were observed in the scaffold structure and examined (Abdollahi Boraei et al., 2020a). After the HNTs are added to the scaffold, most of its peaks are overlapped with gelatin, a phenomenon that has been observed in the previous study (Abdollahi Boraei et al., 2021b). Only peaks of gelatin amide A (3,421 cm^−1^) and O-H stretching of HNTs (3,695 cm^−1^) shifted slightly, indicating successful incorporation of HNTs into the gelatin structure and even reinforcing the possibility of hydrogen bonding interactions between them. With the addition of ZA to the structure and synthesis of Gel-HNT/ZA nanocomposite scaffolds, new peaks appeared, these peaks include the following: P-O bond (1,300 and 1,322 cm^−1^), free hydroxyl group (3,492 cm^−1^), hydroxyl group (2,500–3,300 cm^−1^) (Paris et al., 2015; Chen et al., 2020) that is the reason for the successful addition of ZA to the structure.

**FIGURE 4:**
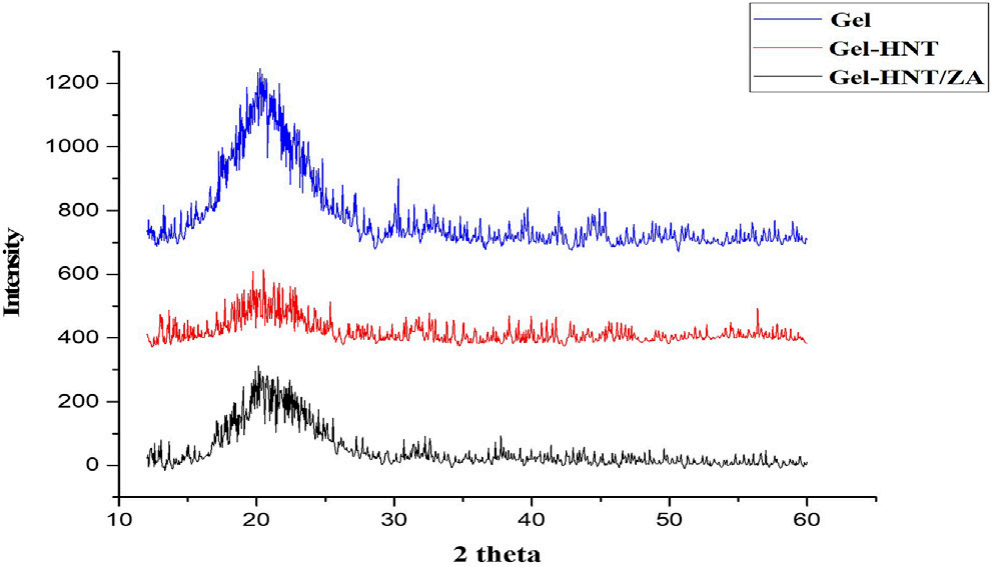
X-ray diffraction pattern of the Gel, Gel-HNT and Gel-HNT/ZA scaffolds.

Figure 4 shows the diffraction pattern of a gelatinous scaffold with a wide peak at 20.25^°^, which is related to the crystalline structure of the gelatin triple helical, which has been reported in several studies (Jalaja et al., 2015). In general, the HNT pattern has many sharp peaks (Liao et al., 2016). A peak at 20.3^°^ is observed which is one of the characteristic peaks of HNT that related to (020/110) plane (Liao et al., 2016). The location of this peak has not changed due to the scaffolding production process, which indicates that the HNT layers do not change and that the HNT and gelatin do not intercalate (White et al., 2012). As a result of the addition of ZA to the Gel-HNT scaffold, a small increase in the intensity of the Gel-HNT scaffold peaks occurred, which could be due to the penetration of ZA into the silicate layers of the HNTs. However, due to the absence of ZA characteristic peaks in the graph, it can be concluded that ZA is not completely crystalline in structure. This may be due to the very low concentration of ZA (˃10^–4^ mol/L) in the structure that is in line with the results of a previous study in which ZA was loaded into a composite structure and coated on a magnesium implant containing strontium (Li et al., 2019).

Table 2 shows the results of the mechanical compressive strength test of scaffolds. As can be seen, the Gel scaffold has the lowest mechanical strength (*σ* = 10.27 ± 1.58 MPa), followed by the Gel-HNT scaffold with (*σ* = 20.92 ± 2.16 MPa) and Gel-HNT/ZA scaffold with (*σ* = 26.18 ± 2.9 MPa). Despite the increase in pore size in Gel-HNT and Gel-HNT/ZA scaffolds, which should have led to a decrease in mechanical strength, the strength of these samples has increased. The reason for this can be related to the presence of HNTs, which is a ceramic with very high mechanical properties, and the presence of HNT in the structure may lead to the bearing and transfer of force throughout the structure and increase the mechanical properties of scaffolds containing HNTs. In several studies, the effect of adding HNT to biopolymers was investigated, all of which showed an increase in the mechanical properties of the resulting structure (Liu et al., 2013; Liu et al., 2015). Increasing the strength of synthesized tissue engineering scaffolds is considered as one of the practical advantages because scaffolds must act as temporary support against stresses and forces until the tissue is completely repaired (Liu et al., 2015).

**TABLE 2:** Compressive strength of the scaffolds.

Sample	Gel	Gel-HNT	Gel-HNT/ZA
Compressive strength (MPa)	10.27 ± 1.58	20.92 ± 2.16	26.18 ± 2.9

### 3.2 *In vitro* Release Study

**FIGURE 5:**
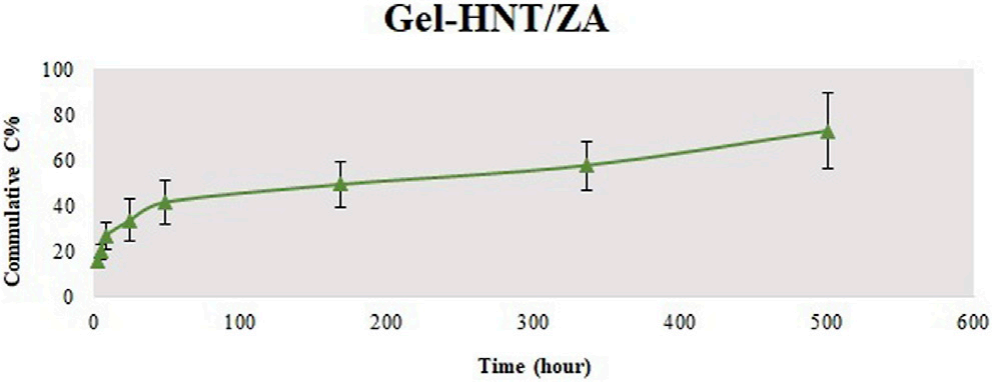
ZA release behavior from the Gel-HNT/ZA.

Recent studies have shown that local delivery of osteogenesis drugs with a controlled behavior can be used as a way to improve large and severe bone defects with low side effects (Boraei et al., 2020). The release behavior of ZA was initiated by a low burst mode and then continued via a stable release with a low rate. Also, the release profile successfully extended to 21 days, meaning proper time to complete *in vitro* osteogenic differentiation of stem cells (Bou Assaf et al., 2019) (Figure 5). Of course, the actual burst release is reduced through bonding between HNT and ZA. In addition, the low crystallinity of the scaffolds with more ZA facilitated the water diffusion into the scaffolds and consequently increased the ZA release from the samples. These results are in accordance with the previous study (Karavelidis et al., 2011), which proved that the ZA release rate is amplified by lowering the crystallinity of the polymer matrix.

### 3.3 hASCs Proliferation and Differentiation

**FIGURE 6:**
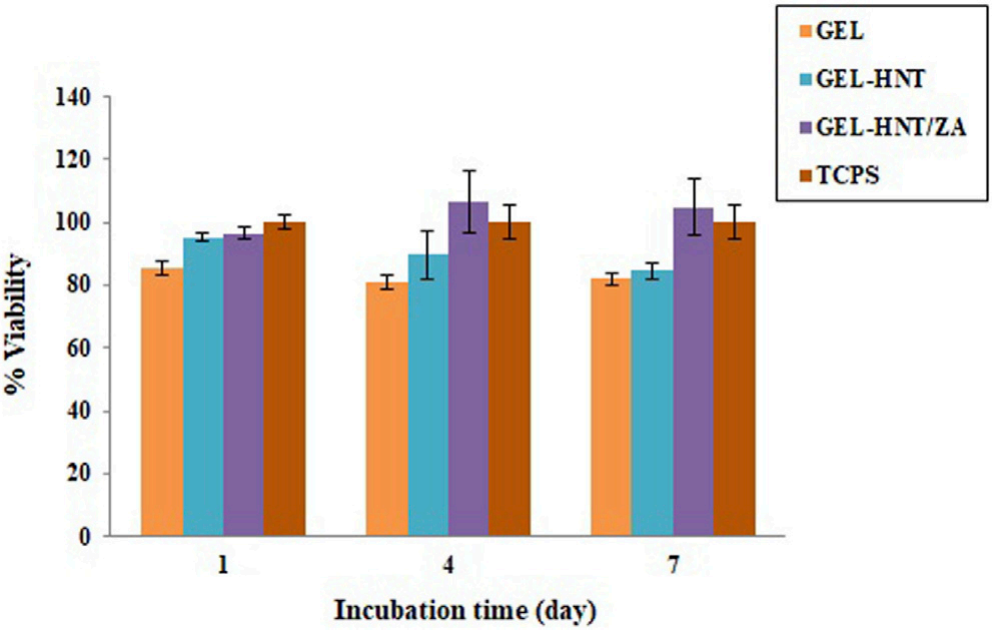
MTT assay of hASCs cultured on the scaffolds (*p* < 0.05).

Cell viability and cytocompatibility of the scaffolds are shown in Figure 6. Human Adipose Stem Cells (hASCs) were cultured onto the samples, and an MTT assay was done. Cell proliferation was investigated after 1, 4, and 7 days. In a study by Davydenko et al., OD in a sample with pure gelatin scaffold was higher than OD of TCPs, which can be considered due to the presence of more sites for cell-matrix interaction. These places are mostly due to the presence of interconnected micron porosity (Davidenko et al., 2016). These results were also observed in this study, which shows the importance of interconnected porous structures. According to the results, both Gel-HNT and Gel-HNT/ZA showed more proliferation than Gel sample during the days of culture, especially in ZA-containing scaffold. By adding HNTs nanorods to the structure, the surface-to-volume ratio increases dramatically, and consequently the cell-matrix interaction increase, which in turn increases cell proliferation and differentiation, which is consistent with previous studies (Ji et al., 2017).

**FIGURE 7:**
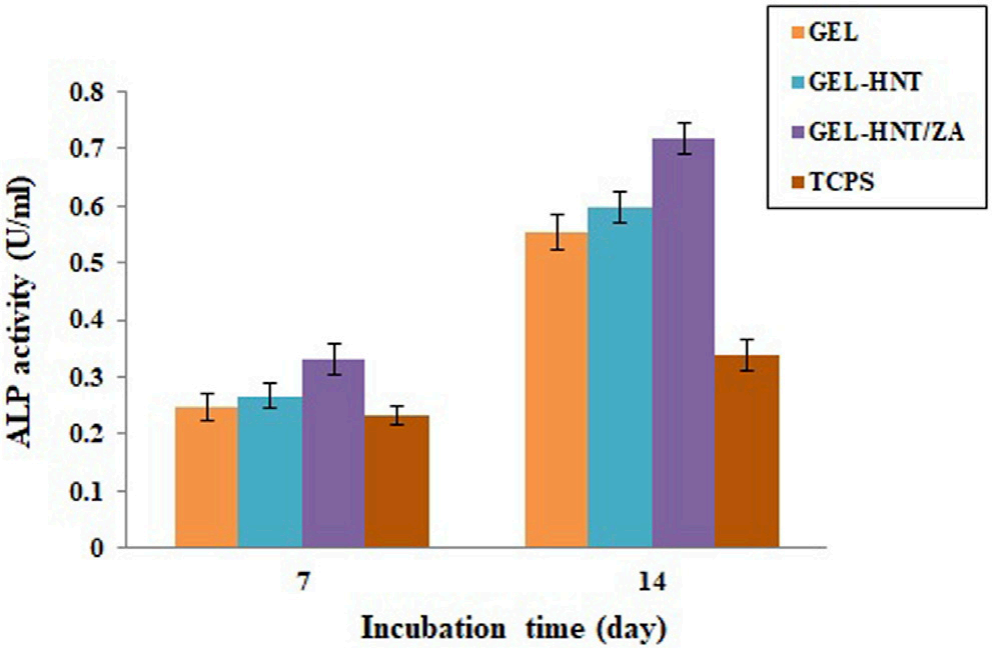
Alkaline phosphatase (ALP) activity of (Gel, Gel-HNT and Gel-HNT/ZA) scaffolds and tissue culture polystyrene (TCPS) at 7 and 14 days, during osteogenic differentiation (*p* < 0.05).

Alkaline phosphatase (ALP) activity assay was used to investigate the osteogenic ability of hASCs. This enzyme is one of the most famous early markers of osteogenesis. There are some researches that demonstrated the role of ALP in osteogenic differentiation (Ardeshirylajimi et al., 2015). The ALP is the final marker for stem cell osteogenic differentiation that promotes the mineralization of calcium (Boraei et al., 2020). This test was performed on scaffolds for 7 and 14 days (Figure 7). The results showed a higher expression of the activity of samples containing ZA on day 14. There was also a slight increase in ALP on day 7. It is important to note that ALP values for all scaffolds were significantly higher than those calculated for TCPs. In addition, the ALP amount is considerably more in the samples including ZA, suggesting the speed up of osteogenic differentiation of seeded hASCs by the scaffold-released ZA. As reported previously, maximum ALP activity is caused by enhancement in the mineralization (Santo et al., 2012).

**FIGURE 8:**
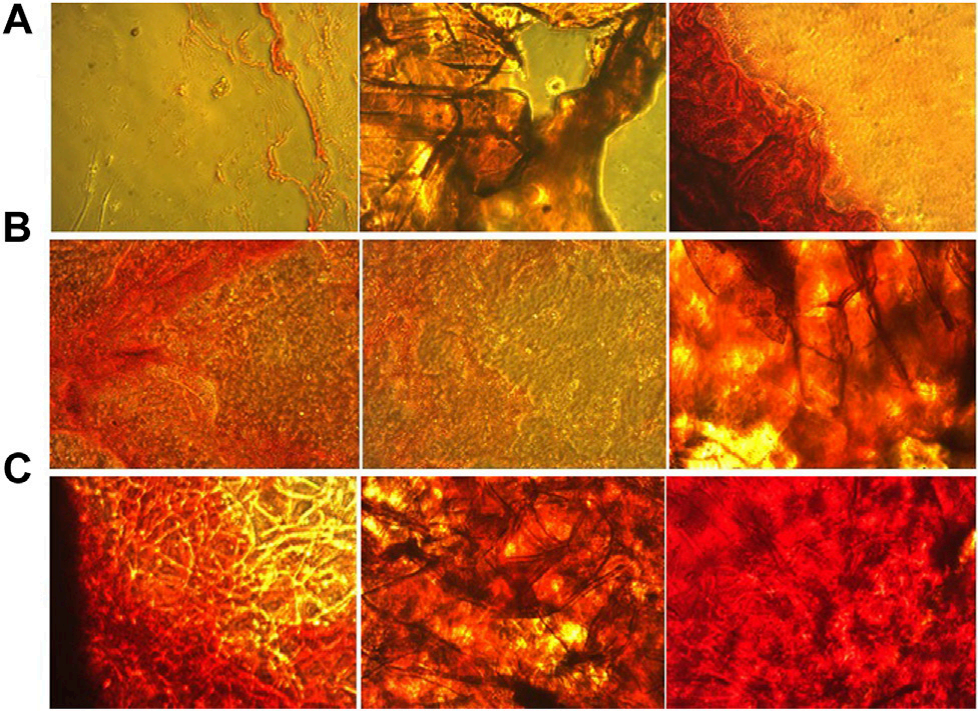
Alizarin red staining of culture hASCs on the **(A)** Gel, **(B)** Gel-HNT and **(C)** Gel-HNT/ZA Scaffolds at 7, 14 and 21 days.

The state of mineralization, which is the formation of inorganic calcium, was also investigated for 7, 14 and 21 days (Figure 8). This state of mineralization (calcium deposition) acts as the late marker of osteogenesis were investigated by Alizarin Red Staining (ARS) (Veernala et al., 2019). In this analysis, the red dots after imaging indicate the amount of calcium deposition at specific times. The amount of deposited calcium was remarkably increased with increasing the incubation time and adding the ZA until 21st day. On day 14, ZA release from the scaffolds caused a large difference in the amount of calcium deposited. The Bone Mineral Density (BMD) and osteogenic ability of ZA have been widely investigated and different mechanisms have been discussed (Pavón de Paz et al., 2019; Jin et al., 2020). In previous studies on ZA, the rate of increase in calcium deposits was observed with increasing time, the amount of calcium islets on the 14th day was significantly higher than the 7th day, which is consistent with our present study that the deposits Calcium has increased over time and the amount of ZA in the scaffold has increased (Raina et al., 2020; Demir-Oğuz and Ege, 2021).

## Conclusion

The present study aimed to inset a novel nanocomposite scaffold to accelerate osteogenesis and deliver enough amount of Zoledronic Acid (ZA) to the bone cells in a suitable and continues manner for osteogenesis. The size of the pores in the scaffold containing ZA reached about 257 microns, which is completely suitable for use in bone tissue engineering. The XRD results showed that the ZA was molecularly scattered in the scaffold structure and addition of ZA diminished the crystallinity of the nanocomposite scaffolds. FTIR and EDS results were proved the successful loading of ZA in the samples. The *in vitro* release assay showed that ZA has displayed a low primary burst release (15%) and then was stable and controlled release up to 21 days (about 49%). The results of Gel-HNT and Gel-HNT/ZA showed higher pore size, porosity, mechanical properties, degradation rate, and water adsorption in comparison with Gel scaffold. Cellular assays on hASCs during 7 and 14 days of culture, demonstrated growth cell viability on all Gel-based scaffolds with and without HNT and ZA. ALP activity assay significantly increased after ZA adding from 0.553 to 0.718. Also, calcium deposition assay completely increased after ZA adding. Hence, Gel-HNT/ZA could be proposed as a capable nanocomposite scaffold for enhancing cell proliferation, osteogenic differentiation, and improving bone regeneration.

## Data Availability

The original contributions presented in the study are included in the article/supplementary material, further inquiries can be directed to the corresponding author.
